# Could the prior development of the retinotopic map account for the radial bias in the orientation map in V1?

**DOI:** 10.1186/1471-2202-16-S1-P27

**Published:** 2015-12-18

**Authors:** Ryan Thomas Philips, V Srinivasa Chakravarthy

**Affiliations:** 1 Department of Biotechnology, Indian Institute of Technology Madras, Chennai, 600036, Tamil Nadu, India

## 

The development of the retinotopic map is presumed to precede the development of the orientation map in V1 in primates. Experimental studies demonstrate a radial bias, wherein radial orientations produce higher activity compared to other orientations [1]. However most traditional models assume isometry in the orientation map developed, i.e. the orientation maps throughout V1 are similar in nature, independent of its retinotopy. In this paper, we propose an activity-dependent model which simulates the development of a radially biased orientation map. To that end we simulate the large-scale development of the retinotopic map, followed by the development of the orientation map in a sub-region of this map. The architecture consists of a Laterally Interconnected Synergetically Self Organizing Map (LISSOM) [2] with 2 layers, representing the retina, and the V1 respectively (see Figure 1A). At each time step, each neuron in V1, combines the afferent activation (*ζ_r1,r2_*) along with its lateral excitations and inhibitions (*η_kl_*) from the previous time step.

(1) ηij(t)=σ(∑r1,r2ζr1,r2 μij,r1r2+ γE∑k,lEij,kl ηkl(t-1)-γI∑k,lIij,kl ηkl(t-1))

The afferent (*μ_ij,r1r2_*), lateral excitatory (*E_ij,kl_*) and lateral inhibitory (*I_ij,kl_*) weights adapt based on a normalized Hebbian mechanism. In order to develop the retinotopic map, the inputs to the retinal layer consists of centered (assumed to be the point of fixation) rectangular bars of varying dilations and rotations as modelled in [3]. The retinotopic map developed, biases the initial configuration of the orientation map (see Figure 1B) since all the bars given during the initial training are centered. For the subsequent refinement of the orientation map, Gaussians of differing orientation and positions (non-centered) are given as inputs to the retinal layer. After training for 4000 iterations (see Figure 1C,D), it is observed that the developed orientation map prefers those orientations which the retinotopy biases it towards, quantified by their corresponding histograms (See Figure 1E,F). As seen from the histogram the area occupied by the region mapping 1250-1500 is larger in the map developed assuming retinotopic bias, compared to that of the map developed assuming isotropy.

**Figure 1:**
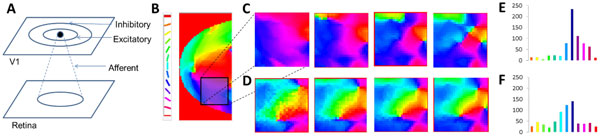
**(A) Schematic representation of the LISSOM architecture**. **(B) **V1 orientation map developed after initial training to establish retinotopy along with its color map. **(C) **Orientation sub-maps, biased by initial retinotopy, at 1000, 2000, 3000, 4000 iterations. **(D) **Orientation sub-maps at 1000, 2000, 3000, 4000 iterations assuming isometry. **(E) **Histogram of the area covered by each of the orientations (color coded) corresponding to (C). **(F) **Histogram of the area covered by each of the orientations (color coded) corresponding to (D).

## Conclusions

A neural activity based model for the development of radially biased orientation maps in V1 is demonstrated.
